# A Label-Free Electrochemical Immunosensor for Detection of the Tumor Marker CA242 Based on Reduced Graphene Oxide-Gold-Palladium Nanocomposite

**DOI:** 10.3390/nano9091335

**Published:** 2019-09-18

**Authors:** Xin Du, Xiaodi Zheng, Zhenhua Zhang, Xiaofan Wu, Lei Sun, Jun Zhou, Min Liu

**Affiliations:** 1 Shandong Provincial Key Laboratory of Animal Resistance Biology, Collaborative Innovation Center of Cell Biology in Universities of Shandong, Institute of Biomedical Sciences, College of Life Sciences, Shandong Normal University, Jinan 250014, Shandong, China; 2 State Key Laboratory of Medicinal Chemical Biology, Key Laboratory of Bioactive Materials of the Ministry of Education, Tianjin Key Laboratory of Protein Science, College of Life Sciences, Nankai University, Tianjin 300071, China

**Keywords:** CA242, tumor marker, rGO-Au-Pd nanocomposite, electrochemical immunosensor, human serum

## Abstract

As a tumor marker, carbohydrate antigen 24-2 (CA242) is a highly accurate and specific diagnostic indicator for monitoring pancreatic and colorectal cancers. The goal of this study was to create a novel label-free electrochemical immunosensor using a nanocomposite glassy carbon electrode for the detection of CA242. Graphene oxide (GO) and polyvinyl pyrrolidone were chosen as the dopants for the preparation of a high-performance reduced-GO-gold-palladium (rGO-Au-Pd) nanocomposite. RGO-Au-Pd was characterized using X-ray diffraction and transmission electron microscopy, revealing that the material exhibited superior electrochemical redox activity and electron transfer ability. The effects of the synthesis method, material concentration, reduction cycle, and pH were investigated to optimize the performance of the immunosensor. As a result of the catalytic activity and biocompatibility of rGO-Au-Pd, the prepared CA242 immunosensor displayed a wide linear range of detection from 0.001 U/mL to 10,000 U/mL with a detection limit of 1.54 × 10^−3^ U/mL and a sensitivity of 4.24 μA (log_10_C_CA242_)^−1^. More importantly, the immunosensor exhibited satisfactory reproducibility and selectivity when detected CA242 in PBS or human serum. The results of our study provide a platform for the development of novel bioassays for use in early cancer diagnosis and promote the application of biosensing technology in the medical field.

## 1. Introduction

Cancer continues to be a major threat to human public health [1]. Currently, tumors of the digestive tract are one of the top ten malignant tumors in the global population; the lethality of these cancers is due, in part, to their insidious onset, early metastasis, low resection success rate, and rapid growth [2,3,4]. Patients are often in the final stages of digestive tract tumors due to the absence of sensitive and specific diagnostic methods. Due to their specificity for cancer cells, tumor biomarkers provide a new means for early detection of both cancer onset and relapse, allowing for timely therapeutic intervention [5,6,7]. Carbohydrate antigen 24-2 (CA242) is a sialylated glycosphingolipid antigen that is clinically used for the diagnosis of malignant tumors of the digestive tract, especially pancreatic cancer and colorectal cancer. Compared with CA19-9 and CA50, the sensitivity can reach 66%~100%, and specificity of CA242 in pancreatic cancer, gallbladder cancer, and digestive tract cancer are higher. CA50 and CA19-9 are susceptible to liver function and cholestasis, so false positives are often seen in benign obstructive jaundice and liver damage [8,9]. Therefore, development of sensitive and reliable methods to detect CA242 will provide tools for early detection of these malignant tumors.

At present, many immunosensors, including amperometric [10], fluorescent [11], colorimetric [12], potentiometric [13], piezoelectric, thermal [14], and radiometric sensors [15], have been created for sensitive detection of tumor markers. All of these assays depend on the reaction between antibodies and application-specific antigens. Among these methods, label-free electrochemical immunosensors have generated substantial interest due to their relatively high sensitivity, cost effectiveness, rapid detection, and lack of need for a secondary antibody [16,17,18].

The sensitivity of an electrochemical immunosensor is significantly affected by the specific surface area and conductivity of the sensing surface [19,20]. Recently, various nanomaterials, including metal nanoparticles [21], carbon materials [22], metal oxides [23] and their derivatives, have been used to modify electrodes so as to amplify the signal and enhance the performance of immunosensors. Among these nanomaterials, hybrid nanocomposites are highly attractive because of their long-term stability and because their enhanced electrical, optical, magnetic, and chemical properties are superior to those of single-component nanocomposites [24].

Gold-palladium (Au-Pd) nanocomposite is a bimetallic nanomaterial that has been widely applied in the field of biosensing because of its biocompatibility, high catalytic ability, and large surface-to-volume ratio [25]. However, the bimetallic material easily aggregates at nanometer dimensions, a characteristic that prohibits more extensive applications. An effective approach to overcome this limitation is to use a carbon material as the template for the preparation of hybrid nanocomposites [26]. Recently, graphene, composed by single layer of carbon atoms, has been widely used in the preparation of electrochemical sensors owing to its remarkable electrical and mechanical properties. Among the reported techniques for the preparation of graphene, the method of acquisition of reduced GO (rGO) from the reduction of graphene oxide (GO) is widely used because GO is highly soluble and environmentally friendly [27,28]. In addition, owing to the excellent performance including catalytic ability, biocompatibility, and electric conductivity of graphene-based nanohybrids, they have been widely used in biosensor applications [29,30,31].

In this study, the rGO-Au-Pd nanocomposite was synthesized using polyvinyl pyrrolidone (PVP) as a protective agent and formic acid as a reducing agent. We then used this nanocomposite as a platform for preparation of a label-free electrochemical immunosensor designed to detect CA242 in human serum. This is the first report of a CA242 immunosensor assay based on a modified rGO-Au-Pd nanocomposite electrode. The goal of this study was to develop a novel platform for use in early diagnosis of cancer and to promote the application of biosensing technology in the medical field.

## 2. Experimental Methods

### 2.1. Chemicals and Reagents

GO was obtained from Xfnano Materials Tech (Nanjing, China). CA242 and anti-CA242 antibodies were obtained from Fitzgerald Industries International. PVP, palladium chloride (PdCl_2_), ascorbic acid, chloroauric acid (HAuCl_4_), dopamine, formic acid (HCOOH), and uric acid were purchased from Sigma-Aldrich (St. Louis, MO, USA). Doubly distilled water was applied to prepare all detection systems.

### 2.2. Electrochemical Measurements

An EG&G 283 Potentiostat–Galvanostat electrochemical workstation equipped with M270 software (Ametek; Berwyn, PA, USA) was used to get electrochemical results. A classic three-electrode system was connected to an electrochemical cell in order to carry out electrochemical measurements. A modified glassy carbon electrode (GCE) whose diameter was 3-mm-diameter was regarded as the working electrode. The reference electrode and the counter electrode were composed of an Ag/AgCl (saturated KCl) electrode and a platinum wire (1-mm diameter), respectively. All electrochemical experiments were performed at room temperature.

### 2.3. Characterization of the Prepared Nanocomposites

A Quanta-200 field emission microscope was used to obtain the transmission electron microscopy (TEM) pictures. X-ray diffraction (XRD) data were obtained from a diffractometer with a D/max-rA and Cu Kα radiation (*λ* = 1.5418, Rigaku, Japan).

### 2.4. Fabrication of Homogenous rGO-Au-Pd Composite

Homogenous rGO-Au-Pd nanocomposite was prepared, with modifications, according to previously reported methods [32]. Briefly, 20 mg GO (0.5 mg/mL) and 80 mg PVP were dissolved in doubly distilled water with continuous stirring for 12 h. Next, 250 μL of 56.4 mM PdCl_2_, 282 μL of 50 mM HAuCl_4_, and 350 μL of HCOOH were added to the solution at room temperature with vigorous stirring (1:1 molar ratio Au to Pd). Due to the aldehyde group, HCOOH is reductive and can reduce the GO to rGO. Additionally, potassium iodide and hexadecylpyridinium chloride were applied as reducing agents instead of HCOOH. The mixture was allowed to react at 95 °C until the color turned from brown to dark. The dark homogeneous solution was cleared by centrifugation at 10,000 rpm, and subsequently a mixture of ethanol and doubly distilled water was applied to wash the sediment at least three times. The re-dispersion was dried in a drying oven to prepare the rGO-Au-Pd composite. For subsequent applications, the composite was resuspended in phosphate-buffered saline (PBS, 2 mg/mL) in a bath sonicator. The rGO and rGO-Pd were prepared using a similar method.

### 2.5. Electrode Modification

**Scheme 1:**
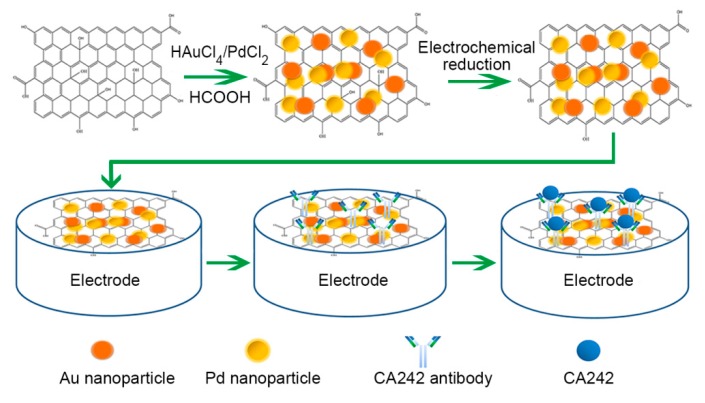
Fabrication of the CA242 immunosensor.

Alumina powders with the diameter of 0.3 and 0.05 μm were used to carefully polish the surface of the GCE in order to remove oxide layers. After that, in order to remove other physically adsorbed substances, the surface of electrode was ultrasonically washed using doubly distilled water and ethanol in turn. The GCE was immediately dried under nitrogen gas. The surface of the GCE was then treated by rGO-Au-Pd film through placing 8 μL of the rGO-Au-Pd suspension (2 mg/mL), followed by drying naturally. The morphological photos of the surfaces of electrodes before and after modified by nanomaterials are shown in Figure S1. The rGO-Au-Pd was completely reduced electrochemically using cyclic voltammetry by (−1.5 V–0 V, 10 cycles) in N_2_-saturated PBS. To obtain rGO-Au-Pd-*anti*-CA242/GCE, 10 μL of 60 μg/mL anti-CA242 was added dropwise, and the electrode was incubated overnight at 4 °C in refrigerator. Aiming to block any remaining sites, the rGO-Au-Pd-*anti*-CA242/GCE was then incubated with 0.1% bovine serum albumin (BSA), yielding rGO-Au-Pd-*anti*-CA242-BSA/GCE (Scheme 1). The rGO-Au-Pd-anti-CA242-BSA/GCE electrode was stored at 4 °C when not in use.

### 2.6. Calculation of the Microscopic Electroactive Areas

The microscopic electroactive areas of prepared modified electrode were calculated according to the equation: Ip = 2.69 × 10^5^AD^1/2^*n*^3/2^γ^1/2^C (Randles–Sevcik) [33]. In the equation, Ip represents the maximal current, A corresponds to the electroactive area of each composite, D relates to the diffusion coefficient of the electroactive material in the experimental solution (about 6.7 × 10^−6^ cm^2^ s^−1^), n is a constant related to the number of electrons transferred, γ (50 mV/s) and C (10 mM) are the scan rate and the concentration of the probe molecule, respectively.

## 3. Results and Discussion

### 3.1. Characterization of Composite Materials

**Figure 1:**
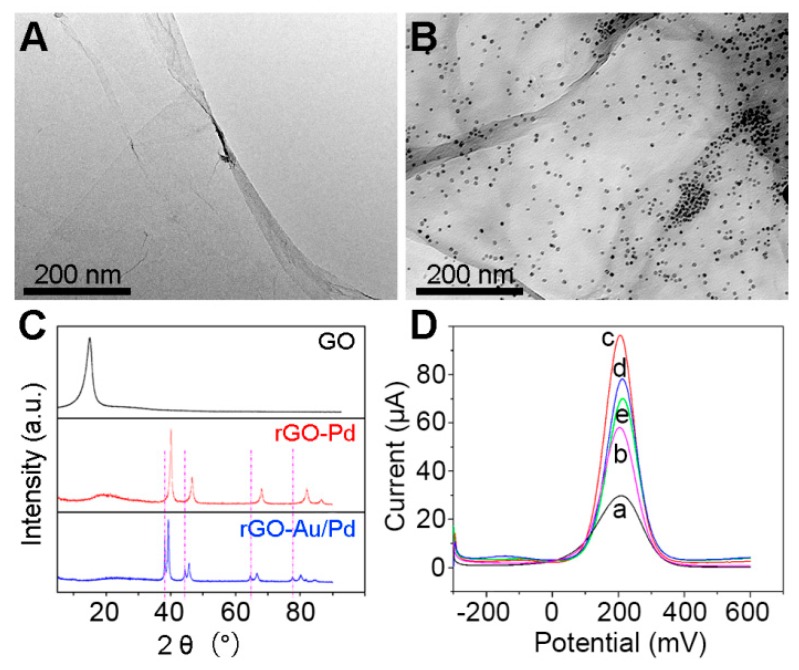
Characterization of the morphology, structure, and electrochemical performance of the nanocomposites. (**A**,**B**) Transmission electron microscopy images of GO (**A**) and rGO-Au-Pd (**B**). (**C**) X-ray diffraction profiles of GO, rGO-Pd, and rGO-Au/Pd. (**D**) Differential pulse voltammetry measurements of bare GCE, GO-Au-Pd/GCE, rGO-Au-Pd/GCE, rGO-Au-Pd-anti-CA242/GCE, and rGO-Au-Pd-anti-CA242-BSA/GCE obtained in 10 mM K_3_[Fe(CN)_6_].

The morphology and structure of the nanocomposites, GO and rGO-Au-Pd, were characterized using transmission electron microscopy. Pure GO demonstrated as a transparent thin film, illustrating that it was entirely exfoliated in the aqueous solution (Figure 1A). The average diameters of small Au nanoparticles (AuNPs) and Pd nanoparticles (PdNPs), which decorated the GO film, were 5 nm (Figure 1B).

The crystal structure of the nanocomposites was detected by X-ray diffraction, and the diffraction results of GO, rGO-Pd, and rGO-Au-Pd were generated (Figure 1C). In the X-ray diffraction pattern of GO, we clearly observed a characteristic (0 0 2) peak at 2 *θ* = 10.6°. After HCOOH and electrochemical reduction treatment, the GO was reduced, as signified by the emergence of a new and broad diffraction peak at 2 *θ* = 22.7° that was generated by the production of rGO. This peak was also observed in the patterns of rGO-Au-Pd and rGO-Pd, demonstrating that the method is suitable for the reduction of GO to rGO [34]. The broad peak of rGO can be ascribed to to a relatively short domain order and the small sheet size of the rGO stacked sheets [35]. In the X-ray diffraction pattern of rGO-Pd, peaks located at 2 *θ* = 40.118°, 46.685°, and 68.119° were respected to the (111), (200), and (220) lattice plane of the Pd nanoparticles, respectively. In the diffractogram of the rGO-Au-Pd, the simultaneous presence of characteristic (111) peaks of metallic gold and palladium located in 2 *θ* = 38.2°, 44.4° and 2 *θ* = 40.1°, 46.7° respectively, is an indication of alloy formation [36]. Furthermore, the elemental mapping and EDX analysis of rGO-Au-Pd also demonstrated the successful preparation of the composite nanomaterial (Figure S2).

Aiming to investigate the fabrication procedures of the CA242 immunosensor, differential pulse voltammetry measurements were performed in 10 mM K_3_[Fe(CN)_6_] (Figure 1D). Current signals for GO-Au-Pd and rGO-Au-Pd were substantially larger than that of bare GCE. Anti-CA242 antibody was coated on the rGO-Au-Pd/GCE, which was indicated with a further reduction in peak size. The current was further decreased after incubating the resulting sensing surface with BSA solution in order to block nonspecific sites; the additional protein hindered electron transfer, resulting in the observed decrease in conductance. These results indicate that the CA242 biosensor was successfully generated.

### 3.2. Characterization of the Electrocatalytic Activities of the Modified Electrodes

**Figure 2:**
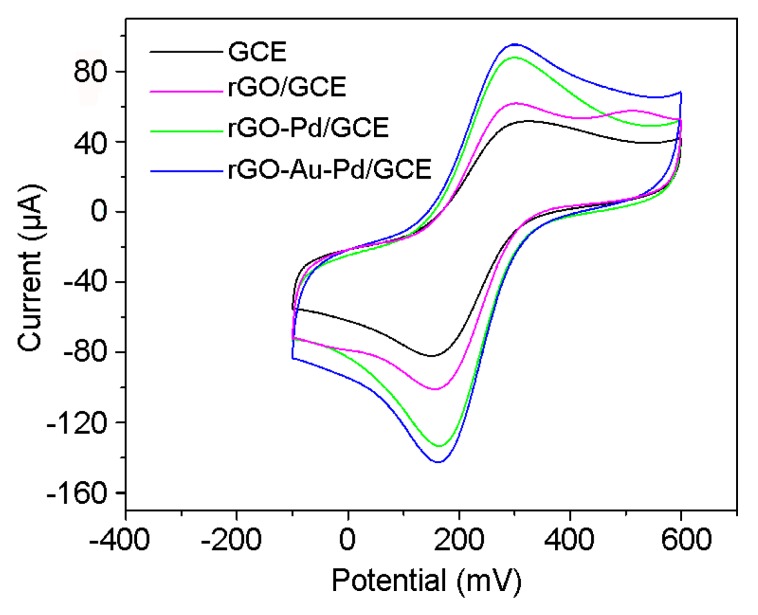
Measurement of the microscopic electroactive areas of GCE, rGO/GCE, rGO-Pd/GCE, and rGO-Au-Pd/GCE. Cyclic voltammograms generated by the indicated electrodes recorded in 10 mM K_3_[Fe(CN)_6_] at a scan rate of 50 mV/s.

The electrical activities of bare GCE, rGO/GCE, rGO-Pd/GCE, and rGO-Au-Pd/GCE were investigated using cyclic voltammetry in a ferricyanide system. The cyclic voltammetry experiments all revealed well-defined redox peaks at about 280 and 170 mV for each modified electrode; these results should be ascribed to the quasi-reversible redox performance of ferricyanide ion in the system (Figure 2). The values of the cathodic peaks of bare GCE, rGO/GCE, rGO-Pd/GCE, and rGO-Au-Pd/GCE were 82.0, 100.9, 133.3, and 142.6 μA, respectively. The rGO-Au-Pd/GCE exhibited the best electrochemical performance due to the synergistic effect of the bimetal nanocomposite (RSD = Figure S3). The calculated microscopic electroactive area of the rGO-Au-Pd/GCE was 1.74-, 1.41-, and 1.07-times higher than that of the bare GCE, rGO/GCE, or rGO-Pd/GCE, respectively. The observed increases in the electroactive area may be due to the excellent electrical conductivity and increased surface area of the rGO-Au-Pd nanocomposite. The above results also proved that the rGO-Au-Pd nanocomposite is suitable for the preparation of a biosensor.

**Figure 3:**
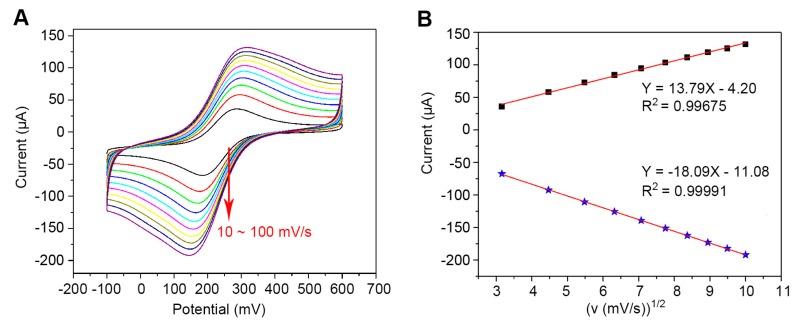
Kinetic analysis of rGO-Au-Pd/GCE indicates that the reaction of the modified electrode is a diffusion-controlled surface reaction. (**A**) Cyclic voltammograms of the sensor in 10 mM K_3_[Fe(CN)_6_] at scan rates ranging from 10 to 100 mV/s. (**B**) Linear fits of the oxidized peak current (Ipa) and reduced peak current (Ipc) versus the square root of the scan rate (v^1/2^).

The kinetics of the modified electrode were explored through studying the effects of the scan rate on cyclic voltammetry. The electrochemical performance of rGO-Au-Pd /GCE was examined in a 10 mM potassium ferricyanide solution with scan rates ranging from 10 to 100 mV/s. The values of maximal current of the redox reaction added linearly with increasing scan rate; additionally, the distance between redox peaks became greater and greater (Figure 3A). Based on the above results, we performed a linear fit about oxidation peak (Ipa) and reduction (Ipc) peak currents related to the square root of the scan rate (*v*^1/2^) (Figure 3B). The ultimate linear equations were determined to be Ipa = 13.79 *v*^1/2^ (mV/s) − 4.20 (*R*^2^ = 0.99675) and Ipc = −18.09 *v*^1/2^ (mV/s) − 11.08 (*R*^2^ = 0.99991). The results from these calculations indicate that the reaction on the surface of immunosensor was a diffusion-controlled surface reaction.

### 3.3. Effect of Experimental Condition on the Performance of the CA242 Immunosensor

**Figure 4:**
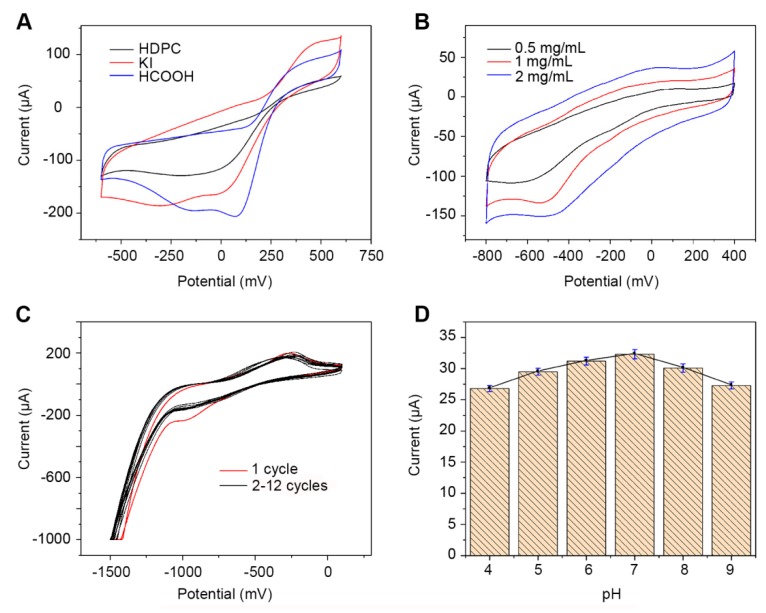
Optimization of experimental conditions used in the generation of the CA242 immunosensor. (**A**) Cyclic voltammograms of the sensor synthesized by different reductants. (**B**) Cyclic voltammograms of the sensor generated by different concentrations of the material. (**C**) Cyclic voltammograms of the sensor generated by different reduction cycles in N_2_-saturated PBS. (**D**) Response currents of the sensor measured at various pH values.

Experimental procedure can influence the electrochemical performance of a modified electrode and, consequently, lead to changes in electrochemical performance and sensing function. The effects of the synthetic method, material concentration, reduction cycle, and pH were investigated in order to optimize CA242 detection. The effect of the synthetic method was analyzed by performing CV in a 5 mM H_2_O_2_ solution to measure the electrochemical performance of rGO-Au-Pd/GCE. Results from this experiment showed that the cathodic peak of H_2_O_2_ appeared at 100 mV, and that the modified electrode exhibited optimal catalytic ability when HCOOH was used as the reductant (Figure 4A). The proportion of Au and Pd used in the synthesis of nanomaterial was also studied, which showed the material had the largest redox current when the radio was 1:1 (Figure S3).The effect of the concentration (Figure 4B) of modified material was analyzed by CV in PBS (Figure 4B). We found that the electrode had the best performance when material was used as 2 mg/mL to modify its surface.

Additionally, the effect of the reduction cycle on electrochemical activity of rGO-Au-Pd/GCE was investigated by cyclic voltammetry in 0.1 M PBS. The resulting spectra showed an obvious cathodic peak as a result of the reduction of rGO-Au-Pd/GCE in the first cycle ranging from −1.5 to 0 V in N_2_-saturated PBS (Figure 4C); these data demonstrate that rGO-Au-Pd was reduced completely [37]. The electrochemical performance of rGO-Au-Pd/GCE tended to be stable after four reduction cycles; however, 10 cycles were used in our experiment to ensure the complete and stable reduction of rGO-Au-Pd/GCE.

The pH of a solution plays a vital role in the determination of protein structure and function; therefore, the effect of pH on the response current of 5 U/mL CA242 in 0.1 M PBS was determined. Response currents increased as pH values were increased from 4.0 to 7.0, reaching a maximum when pH was 7.0, and decreasing at higher values (Figure 4D). At pH 7.0, the anti-CA242 antibody may have more activity, therefore interacting more readily with CA242. To achieve optimized results, PBS at pH 7.0 was applied in subsequent experiments.

### 3.4. Characterization of the Analytical Performance of the Immunosensor for Detection of CA242 in PBS

**Figure 5:**
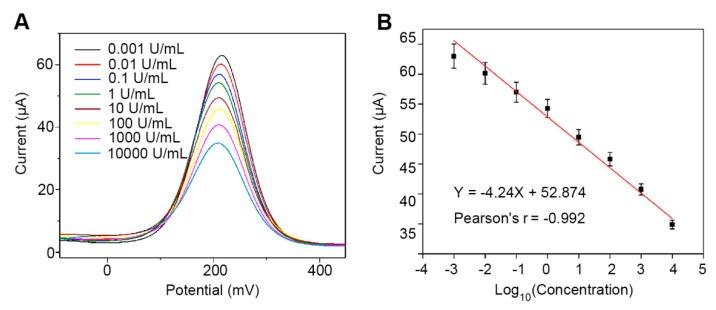
The immunosensor has a wide linear range for detection of CA242 in PBS. (**a**) Differential pulse voltammetry detection of varying concentrations of CA242 ranging from 0.001–10,000 U/mL. (**b**) Calibration curve showing the sensitivity of the immunosensor for the detection of CA242. Error bars represent the mean ± standard deviation (*n* = 5).

Under optimized experimental conditions, the analytical performance of the proposed CA242 immunosensor was determined in 10 mM K_3_[Fe(CN)_6_] solution using varying concentrations of CA242. Differential pulse voltammetry measurements showed the peak current signal, which was typical of the immunosensor, decreased with increasing concentrations of CA242 (Figure 5A). This decrease in performance is likely attributable to the additional protein which inhibited the electron transfer capacity of the electrode.

The proposed CA242 immunosensor exhibited a broad linear range of performance from 0.001 to 10,000 U/mL; the limit of detection was 0.067 mU/mL with a signal-to-noise ratio of 3. The peak differential pulse voltammetry current showed a linearly relation between the CA242 concentration in the linear range. The linear regression equation was calculated as I = −4.24 log_10_C_CA242_ + 52.874 with a Pearson coefficient of −0.992 (Figure 5B). Comparison of the linear range, limit of detection, and sensitivity of this novel immunosensor with previously described immunosensors showed that the prepared CA242 immunosensor exhibited a markedly wider linear range of detection (Table 1).

### 3.5. The CA242 Immunosensor Reliably Detects CA242 in Human Serum

**Figure 6:**
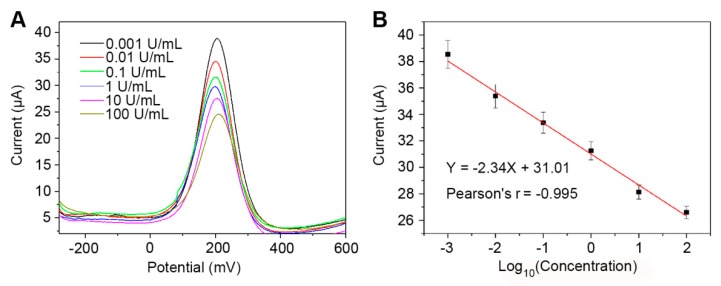
The immunosensor has a wide linear range for detection of CA242 in human serum. (**A**) Differential pulse voltammetry detection of different concentrations of CA242 ranging from 0.001–100 U/mL. (**B**) Calibration curve showing the sensitivity of the immunosensor for the detection of CA242. Error bars represent the mean ± standard deviation (*n* = 3).

To evaluate the reliability of the CA242 immunosensor, varying concentrations of CA242 in human serum samples diluted by 0.1 M PBS (pH 7.4) were analyzed by differential pulse voltammetry (Figure 6A). Each human serum sample was examined three times, and the results for concentrations ranging from 0.001 to 100 U/mL were analyzed by linear regression. The calibration curve varied from 1.8% to 2.7% (*n* = 3, Pearson coefficient = −0.995); these results indicate the excellent reproducibility and reliability of the prepared biosensor for the detection CA242 in human samples (Figure 6B).

### 3.6. The CA242 Immunosensor Exhibits High Reproducibility, Selectivity, and Stability

**Figure 7:**
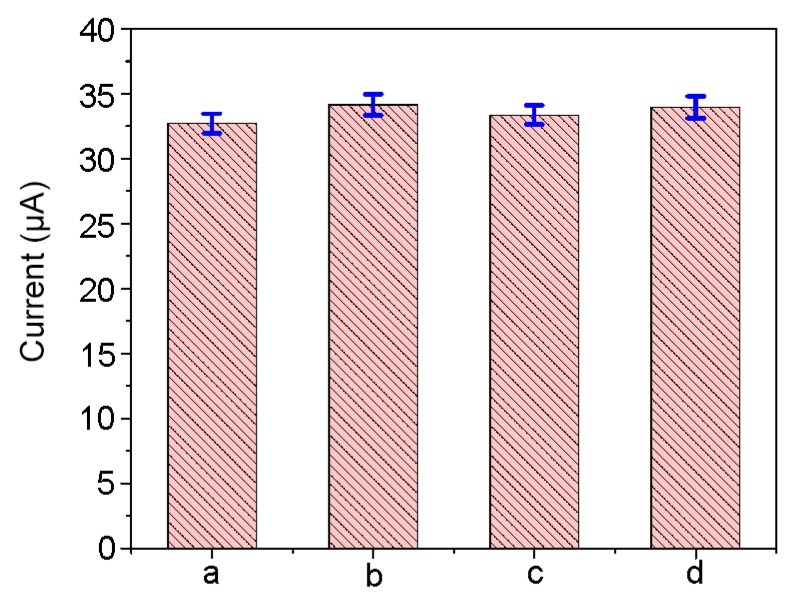
Results obtained using the CA242 immunosensor are highly sensitive and reproducible. Statistics for current responses obtained from five parallel experiments using 5 U/mL CA242 in pure human serum (**a**) and with 0.15 mM ascorbic acid (**b**), 0.5 mM uric acid (**c**), or 0.15 mM dopamine (**d**).

The reproducibility of the CA242 immunosensor was investigated through analyzing the results obtained from five parallel experiments using 5 U/mL CA242. The relative standard deviation was 2.3%, a result that demonstrates the excellent reproducibility of the CA242 biosensor (Figure 7a). Ascorbic acid, uric acid, and dopamine are the electroactive components in human blood that have the greatest interference in electrochemical detection assays. Therefore, to evaluate the selectivity of the immunosensor, interference from ascorbic acid, uric acid, and dopamine was tested with the modified electrode. The relative standard deviations in the presence of 0.15 mM ascorbic acid (Figure 7b), 0.5 mM uric acid (Figure 7c), and 0.15 mM dopamine (Figure 7d) were 4.4%, 2.0%, and 3.8%, respectively; these results indicate that the modified electrode shows exceptional specificity. When not in use, the immunosensor would be stored at 4 °C in PBS (pH 7.0). After one month of storage, 80.6% of the initial current response was retained (Figure S5), suggesting that the prepared sensor also exhibits exceptional stability.

## 4. Conclusions

A label-free immunosensor for detection of CA242 was developed based on an electrochemical redox-active nanocomposite of rGO-Au-Pd. The prepared electrodes showed favorable electrochemical redox activity at 200 mV in a 10 mM K_3_[Fe(CN)_6_] solution, which was used as the current signal for detection. This immunosensor has a wide range of linear detection, excellent sensitivity, and a low detection limit (*S/N* = 3). Overall, this immunosensor performs better than most previously described tumor marker sensors. Additionally, the detection results obtained from analysis of human serum show that this immunosensor exhibited exceptional relative standard deviation and linear equation values. This synthetic method and sensing substrate could be employed for the analysis of other biomarkers and, importantly, provides a model for the creation of other label-free electrochemical immunosensors.

## Figures and Tables

**Table 1:** Comparison of selected electrochemical sensors used in tumor marker detection.

Material	Target	Linear Range (U/mL)	Detection Limit (U/mL)	Sensitivity (μA (log10 c)^−1^)	Electrochemical Method	Reference
Chit-Au ^a^/Ab ^b^1/Ag ^c^/Zn-CP ^d^-Ab2	CA242	1–150 U/mL	0.40	5.03	SWV	[38]
Au/PDDA/PTCA/CNTs	CA242	0.010 to 10 ng/mL	3.8 pg/mL	/	DPV	[39]
SA-Pb^2+^-GO hydrogel	CA242	0.005–500	0.067	32.98	SWV	[40]
Chitosan/Ab1/Ag/Ab2-Ag/g-C3N4 ^e^	CA199	0.005–50	1.2 × 10^−3^	1.46	DPV	[41]
AuAg hollow nanocrystals	CA199	1–30	0.23	0.05	DPV	[42]
Gold nanostructure	CA125	10–100	5.5	0.16	DPV	[43]
Fc–GO ^f^	CA153	0.05–20	0.015	0.34	DPV	[44]
rGO-Au-Pd	CA242	0.001–10,000	1.54 × 10^−3^	4.24	DPV	this work

^a^ chitosan-gold nanocomposites, ^b^ antibody, ^c^ antigen, ^d^ zinc ions doped chitosan-poly(acrylic acid) nanospheres, ^e^ silver-functionalized g-C3N4 nanosheets, and ^f^ ferrocene hybridized graphene oxide.

## Supplementary Materials

The following are available online at https://www.mdpi.com/2079-4991/9/9/1335/s1, Figure S1: The morphological photos of the surfaces of electrodes that before (A) and after (B) modified by Au-Pd-rGO nanomaterials. Figure S2: The elemental mapping and EDX analysis of rGO-Au-Pd., Figure S3: Optimization of experimental conditions of the proportion (3:1, 2:1, 1:1, 1:2, 1:3) of Au and Pd used in the synthesis of nanomaterial. Figure S4: The study of the reproducibility (RSD = 1.86 %) and stability of the prepared biosensor.
