# Supplementary material for: A Label-Free Electrochemical Immunosensor for Detection of the Tumor Marker CA242 Based on Reduced Graphene Oxide-Gold-Palladium Nanocomposite
Source: Nanomaterials (Basel). 2019 Sep 18;9(9):1335. doi: 10.3390/nano9091335 (PMC6781068; doi:10.3390/nano9091335)
Supplement: Supplementary file 1 [file nanomaterials-09-01335-s001.pdf]

nanomaterials

Supplementary Materials

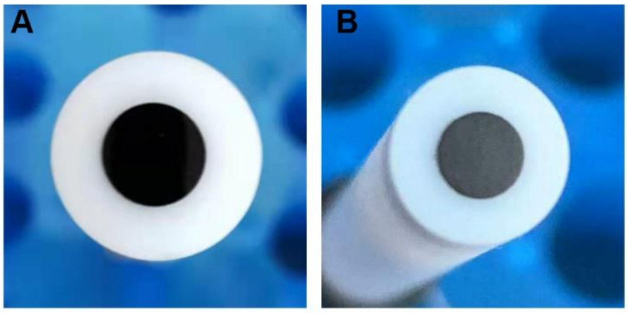

Figure S1. The morphological photos of the surfaces of electrodes that before (A) and after (B) modified by Au-Pd-rGO nanomaterials.

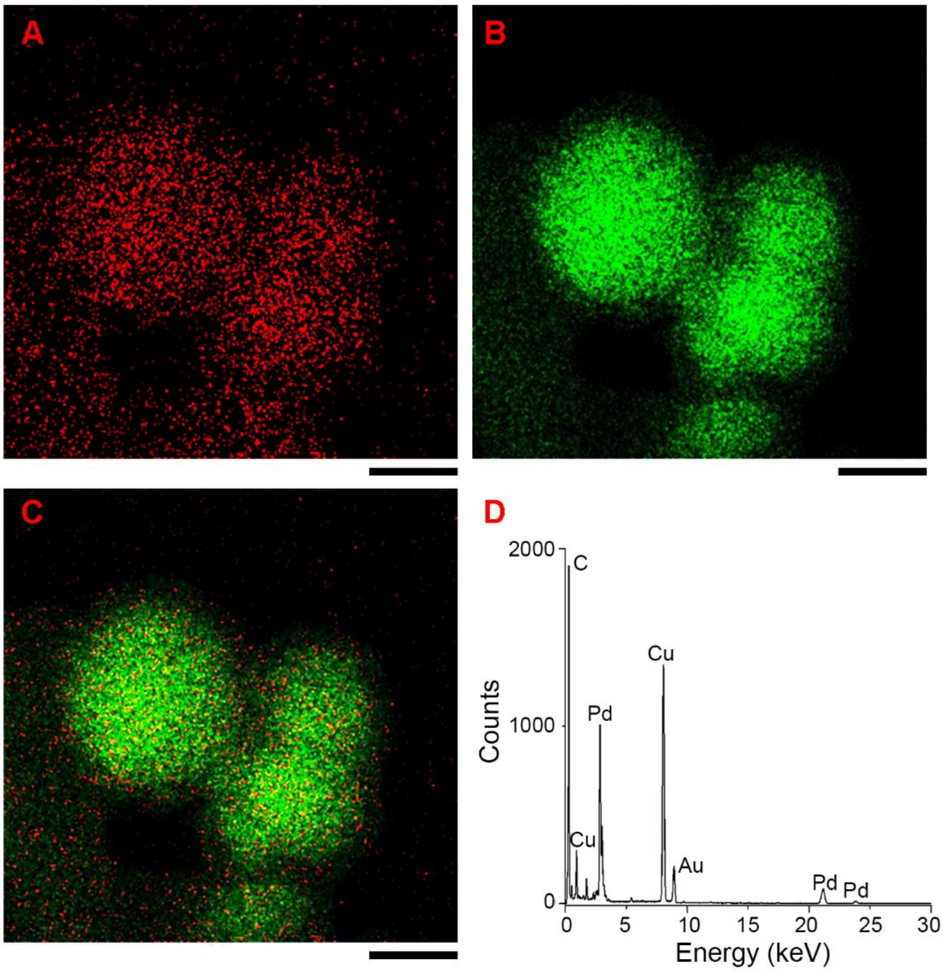

Figure S2. The elemental mapping and EDX analysis of rGO-Au-Pd. The mapping of Au (A), Pd (B) and the merge mapping of Au-Pd elements. (Scale bar, 10 nm). (D) The EDX analysis of rGO-Au-Pd.

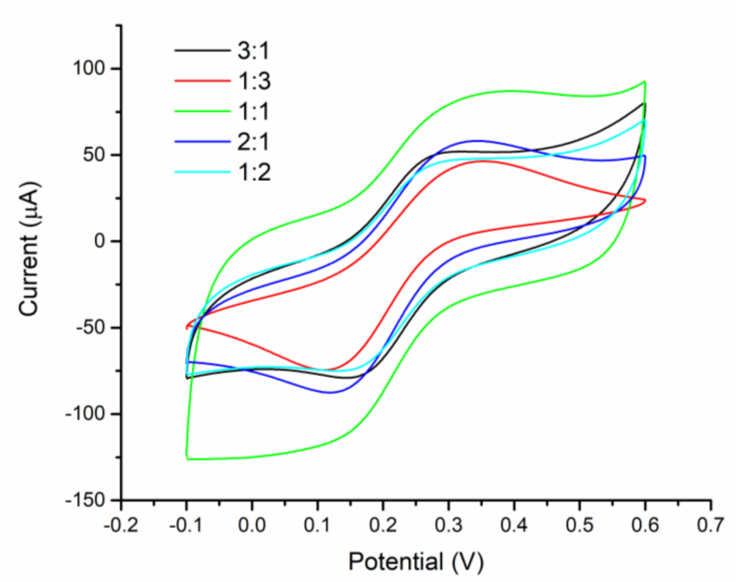

Figure S3. Optimization of experimental conditions of the proportion (3:1, 2:1, 1:1, 1:2, 1:3) of Au and Pd used in the synthesis of nanomaterial.

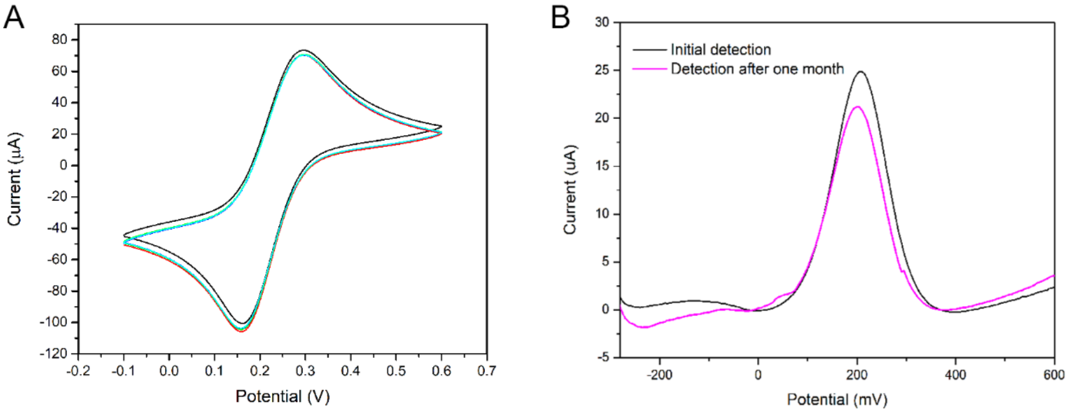

Figure S4. The study of the reproducibility (RSD=1.86 %) and stability of the prepared biosensor.
